# Glutathione S-Transferase Polymorphisms and Clinical Characteristics in Autism Spectrum Disorders

**DOI:** 10.3389/fpsyt.2021.672389

**Published:** 2021-06-25

**Authors:** Vanja Mandic-Maravic, Marija Mitkovic-Voncina, Marija Pljesa-Ercegovac, Ana Savic-Radojevic, Miroslav Djordjevic, Marko Ercegovac, Tatjana Pekmezovic, Tatjana Simic, Milica Pejovic-Milovancevic

**Affiliations:** ^1^Institute of Mental Health, Belgrade, Serbia; ^2^Faculty of Medicine, University of Belgrade, Belgrade, Serbia; ^3^Institute of Medical and Clinical Biochemistry, Belgrade, Serbia; ^4^University Children's Hospital, Belgrade, Serbia; ^5^Clinic of Neurology, Clinical Center of Serbia, Belgrade, Serbia; ^6^Institute of Epidemiology, Belgrade, Serbia; ^7^Serbian Academy of Sciences and Arts, Belgrade, Serbia

**Keywords:** GST, clinical characteristics, intellectual functioning, adaptive functioning skills, autism spectrum disorders

## Abstract

**Background:** Autism spectrum disorders (ASD) are a heterogeneous group of developmental disorders, with different levels of symptoms, functioning, and comorbidities. Recent findings suggested that oxidative stress and genetic variability in glutathione S-transferases (GSTs) might increase the risk of ASD development. We aimed to determine whether GST polymorphisms influence the severity of symptoms as well as the cognitive and adaptive abilities in children with ASD.

**Methods:** The sample included 113 ASD cases. All participants were genotyped for GSTA1, GSTM1, GSTT1, and GSTP1 polymorphisms. The clinical characteristics were determined with Autism Diagnostic Interview-Revised (ADI-R) in all of the participants. In non-verbal participants, we explored the adaptive functioning using the Vineland Adaptive Behavior Scale II, while in verbal participants, we used the Wechsler Abbreviated Scale of Intelligence (WASI).

**Results:** It was shown that the GSTA1^*^CC genotype was a predictor of a lower non-verbal communication impairment as well as of a lower chance of having seizures during life. *GSTM1-active* genotype predicted a higher adaptive functioning. The predictive effect of *GSTA1, GSTM1*, and *GSTT1* genotype was moderated by exposure during pregnancy (maternal smoking and medication). The GSTP1^*^IleIle genotype was significantly associated to a better cognitive functioning in children with ASD.

**Conclusion:** Besides the complex gene-environment interaction for the specific risk of developing ASD, there is also a possible complexity of interactions between genetic and environmental factors influencing the level of symptoms and impairment in people with ASD. Detoxification and antioxidant enzymes, such as GSTA1, might contribute to the core of this complexity.

## Introduction

Autism spectrum disorders (ASD) are a heterogeneous group of disorders with key clinical features being deficits in social communication and restricted, repetitive patterns of behavior, interest, or activities (1, 2). As most of the developmental disorders, ASD are also considered complex genetic disorders and might be defined as clinical manifestations developed as a consequence of different factors, including genetic, environmental factors, as well as their interaction (3–5).

Recent data have shown the possible role of redox imbalance and oxidative stress in ASD (6–9). Oxidative stress exists when there is a dynamic imbalance between the formation of prooxidants and antioxidant capacity of the cell. Redox homeostasis is a balance between the degree of oxidation and reduction and it plays an important role in numerous signaling cascades, including those related to proliferation, inflammatory response, and apoptosis (10–12).

A number of studies so far have shown the possible connection between oxidative stress and ASD (13–15). Glutathione S-transferases (GSTs) have a very important role in antioxidant defense mechanisms by performing the detoxification of xenobiotics and inactivation of a large number of endogenous oxidative stress products (16, 17). All of the four main citosolic classes of GSTs—alpha (GSTA), mi (GSTM), pi (GSTP), and teta (GSTT)—exhibit genetic polymorphism, which can lead to an altered detoxification and oxidative stress defense capacity in certain tissues (18, 19). In relation to that, individuals with an altered GST isoenzyme profile are more prone to the delevopment of different disorders, including psychiatric and developmental disorders. Indeed, several studies have shown a possible role of GST polymorphisms in ASD development (8, 9, 20, 21).

So far, studies have shown that the carriers of the *GSTM1-null* genotype have a higher risk of ASD development (20, 21). A study performed in 2015 has shown no correlation of specific individual GSTP1, GSTT1, and GSTM1 polymorphisms, but there was a significant combined effect between the *GSTP1* and *GSTT1* genotypes in relation to an increased risk of ASD development—in children heterozygous for the *GSTP1* Ile105Val polymorphism—the risk of developing ASD was significantly higher in those with the null *GSTT1* genotype (22). In our previous study, the results have shown a significant effect of the *GSTM1-active* genotype in decreasing the risk of ASD and for the GSTA1^*^CC genotype in increasing the susceptibility to ASD (8). Also, when exploring genotype-genotype interactions, the combination of *GSTM1-active* and *GSTT1-active* as well as combined *GSTT1-active* and GSTP1^*^llelle genotypes decreased the risk of ASD. However, an increased risk of ASD was observed if a combined *GSTM1-active* and GSTP1^*^llelle genotype was present (8). In contrast to these results, Bowers et al. have not recognized the significance of GST polymorphic expression as an individual risk factor for ASD (23).

Interestingly, the research data on the effect of GST polymorphisms on the clinical manifestations of ASD are rather scarce. Two recent studies performed by El Ansary et al. in 2018 and 2020 have examined the predictive value of different biomarkers related to mitochondrial functioning and oxidative stress, in terms of ASD *vs*. healthy controls differentiation. Both studies have shown that the decrease of the GSH/GST ratio was highly predictive of ASD diagnosis. The scale used for the clinical differentiation was the Childhood Autism Rating Scale (CARS) (24, 25). The predictive value of GST levels was 91% (25), but the biomarkers related to oxidative stress were not distinctive for the level of ASD symptoms when measured by CARS (mild to moderate vs. severe ASD) (24). The conclusion of these studies was that, although highly predictive for ASD diagnosis, the GSH/GST ratio as a biomarker was not significantly associated with ASD severity (24).

Besides the level of severity of specific ASD symptoms, it is very important to determine the level of intellectual and adaptive functioning of persons with ASD (26), since these characteristics might be very important for everyday functioning (27, 28). The prevalence of intellectual disability (ID) in ASD is high, with varying results from different studies−16.7–84% (26). Several studies pointed out the connection of oxidative stress and cognitive functioning (29, 30). A study that investigated oxidative stress biomarkers hypothesized that oxidative stress is also linked to early aging in individuals with ID (31, 32).

The studies which explored the role of GSTs in ID are also scarce. A research conducted in 2008 examined GSTM1, GSTT1, and GSTP1 polymorphisms in a cohort of children, along with the measurement of prenatal exposure to p,p′-DDT [2,2-bis(p-chlorophenyl)-1,1,1-trichloroethane], in relation to their cognitive functioning at the age of four. It was shown that children carriers of the GSTP1^*^Val105 allele were at a higher risk of the adverse cognitive functioning effects of prenatal p,p′-DDT exposure (33). Also, a recent study has shown that female medulloblastoma survivors with GSTP1 polymorphism, both G313A (rs1695) and C341T (rs1138272), have an increased vulnerability to deficits in core cognitive skills, IQ, and everyday functioning (34).

Comorbidity, the presence of two or more disorders in one individual, is an important issue associated with a more severe course of illness (35). There is a number of psychiatric comorbidities in ASD, such as anxiety and mood disorders, obsessive compulsive disorder, attention deficit hyperactivity disorder (ADHD), and oppositional defiant disorder (36). Children with ASD exhibit disturbances in gastrointestinal physiology as well, such as increased intestinal permeability and microbiota alterations (37).

One of the most significant comorbidities in ASD is epilepsy. The prevalence of epilepsy in ASD varies across studies, but one of the most recent reviews shows that the period prevalence of this disorder in ASD is 12% (38). The prevalence of epilepsy in ASD has been very different in the literature so far, possibly due to the heterogeneity of the study groups and different definitions (39). When it comes to oxidative stress and epilepsy, there is evidence that oxidative stress might play a role in both seizure initiation and progression of epilepsy (40, 41).

Taken together, it is possible that oxidative stress and specific GST genotypes might be associated with the severity of symptoms in ASD, which means more impairment in verbal and non-verbal communication and more repetitive and stereotyped behavior as well as lower adaptive functioning. Children with more severe symptoms and lower functioning might be in need of medication and/or hospitalization more often in order to perform a detailed diagnostic process and determine the most adequate pharmacotherapy. The severity of symptoms is also correlated with the presence of comorbidities, such as seizures and ID. Furthermore, besides defining the possible effect of genetic factors related to oxidative stress in ASD, it is also important to determine the possible effect of environmental factors, such as prenatal exposure (42).

Our study aimed to determine whether GST gene polymorphisms are associated with the severity of ASD symptoms, intellectual and adaptive functioning of persons with ASD, as well as, other clinical characteristic, such as the presence of seizures, the need for medication, and inpatient diagnostics and treatment.

## Method

### Study Population

The study was performed as a cross-sectional study. The study group involved 113 ASD patients (92 males, 21 females, 9.36 ± 5.88 years old), included as consecutive referrals and treated as outpatients and inpatients at the Institute of Mental Health, Belgrade, Serbia. The inclusion criterion for the case group was the presence of any of the ASD. The diagnosis was verified by the ICD-10 criteria (43), confirmed by a child psychiatrist with an experience in diagnosing ASD. The evaluation was done through a clinical interview with the parent and examination of the child. Besides the clinical interview and criteria, the diagnosis was verified by the Autism Diagnostic Interview-Revised (ADI-R) (44) conducted by a trained child psychiatrist.

### Instruments

*Autism Diagnostic Interview-Revised (ADI-R*) (44). ADI-R is a standardized semi-structured parent/caregiver interview created for the assessment of signs of ASD. It comprises 93 items, and evaluates the child's early development, development of language, functioning of language and communication, loss of speech and motor skills, social development and play, interests, and behavior. The description for each item given by the parent/caregiver is made for childhood (ever) and current behavior. Specific items describing social reciprocity, communication, and restricted, repetitive, and stereotyped behavior (RRSB) are used to reach the scores for these three domains (ADI-R A, ADI-R B, and ADI-R C score, respectively). In this study, the interview was administered by a certified child psychiatrist. Higher ADI-R scores account for more severe symptoms—greater impairment.

*Vineland Adaptive Behavior Scale, Second Edition (Vineland-II)* (45) was used for the assessment of everyday adaptive behavior in non-verbal patients. The questions were answered by parents or caregivers and were related to the description of the patient's adaptive behavior. The instrument assesses adaptive behavior in the domains of Communication (C: receptive, expressive, and written communication skills), Daily Living Skills (DLS: personal behavior as well as domestic and community interaction skills), Socialization (S: play and leisure time, interpersonal relationships, and various coping skills), and Motor Skills (MS: gross and fine), providing the composite score (Vineland-II score) which summarizes the patient's skills in all four domains. It was administered by certified child psychologists. Higher Vineland scores mean better functioning.

*Wechsler Abbreviated Scale of Intelligence-WASI* (46) was used for the assesment of intellectual functioning in verbal patients. It comprises three traditional IQ scores—verbal, manipulative, and total. It is performed for ~30 min. It was administered by certified child psychologists.

*Sociodemographic and exposure questionnaire*. The questionnaire was created specifically for the current study. Besides the basic sociodemographic information, our questionnaire explores the different types of prenatal exposures as well as perinatal complications in the participants of the study. The questionnaire was used to gather information specifically on mothers' smoking during pregnancy and taking medication during pregnancy, which we explored as potential moderators.

Besides the aforementioned interviews and scales, we have taken information on other potentially important parts of clinical characteristics and treatment before performing the study. The data of interest for this study was the number of hospitalizations and information on pharmacotherapy, as well as, seizures. It is important to note that the data on seizures has taken into account having any seizures during life, not including febrile convulsions, without the definite diagnosis of epilepsy.

### DNA Isolation and Genotyping

Total DNA was isolated from 200 μl of the whole peripheral blood using the *QIAamp DNA Blood Mini Kit* (*Qiagen, Chatsworth CA, USA*), in accordance with the manufacturers' protocol. Genotyping was performed blinded to the case-control status. Blinded quality control samples were applied for the validation of the genotyping procedures. Concordance for the blinded samples was 100%. All of the assays included positive and negative controls. All primers used are synthesized and bought from *Metabion International AG* (*Planegg, Germany*) (47).

### The Genotyping of *GSTM1* and *GSTT1*

Multiplex polymerase chain reaction (PCR) method of Abdel-Rahman et al. (48) was done for assessing the presence of amplified PCR products of GSTM1: 215 bp, *GSTT1*: 481 bp, as well as the housekeeping gene *CYP1A1*: 312 bp, which was applied as an internal control. It is important to emphasize that the assay does not make a distinction between heterozygous or homozygous wild-type genotypes. Therefore, it notes only the presence (at least one allele present, homozygote or heterozygote—*GSTM1-active* and *GSTT1-active* genotype, respectively) or the absence (complete deletion of both alleles, homozygote—*GSTM1-null* and *GSTT1-null* genotype, respectively) of the specific genotype. PCR products were visualized on *Chemidoc (Biorad, Hercules, CA, USA*).

### The Genotyping of GSTA1^*^C69T (rs3957357)

The analysis of the SNP GSTA1^*^C69T (rs3957357) was performed using PCR-restriction fragment length polymorphism (RFLP) by the method of Ping et al. (49). A 400 bp fragment was amplified and subjected to overnight incubation at 37°C with enzyme *EarI* (*Thermo Fisher Scientific, Waltham, Massachusetts, USA*). Digested products (*GSTA1-*CC: 400 bp, *GSTA1-*CT: 400 + 308 + 92 bp, and *GSTA1-*TT: 308 +92 bp) were visualized on *Chemidoc (Biorad, Hercules, CA, USA*).

### The Genotyping of GSTP1^*^Ile105Val (rs1695)

For the assessment of SNP polymorphism *GSTP1*-*Ile105Val*, TaqMan® SNP Genotyping Assays (*Life Technologies, Applied Biosystems, Carlsbad, CA, USA, assay ID: C__3237198_20*) was performed for amplifying and detecting the respective SNP alleles in the purified genomic DNA samples, complying to the manufacturers' instructions. DNA concentration and purity were analyzed spectrophotometrically using *GeneQuantpro (Biochrom, Cambridge, England)*. The presence of the *GSTP1-Ile/Ile* genotype was defined as *GSTP1-wild* type, whereas the presence of the *GSTP1-Ile/Val* or *GSTP1-Val/Val* genotype as *GSTP1-variant* genotype.

### Statistical Analysis

Data were processed using multiple linear regression, with GST genotypes as predictors and clinical characteristics as outcomes, controlling for other GST genotypes and subject's age. Moderation analysis, with genotypes as predictors, maternal medication use and smoking in pregnancy as moderators, clinical characteristics of subjects as outcomes, and other genotypes and age as covariates, was conducted in macro PROCESS for SPSS, based on ordinary least square regression within the path analysis, and using the bootstrap confidence intervals (50).

## Results

The descriptive parameters for the continuous and categorical variables measured in our study are shown in Tables 1, 2.

**Table 1:** Descriptive parameters of continuous variables.

**Continuous variables**	**Total *N***	**Minimum**	**Maximum**	**Mean**	**Std. Deviation**
ADIR_age	113	2.50	30.00	9.2814	5.82426
ADIR A total	113	0.00	30.00	21.6726	6.01255
ADIR B verbal	35	2.00	22.00	16.5429	4.92498
ADIR B non-verbal	78	4.00	14.00	10.9359	2.51406
ADIR C total	103	0.00	12.00	5.8544	2.59490
ADIR D total	104	1.00	5.00	3.0192	1.07920
Wasi block design raw score	19	1.00	61.00	15.5789	19.10298
Wasi block design T score	19	22.00	66.00	39.1579	11.66316
Wasi matrix reasoning raw score	19	1.00	32.00	14.1053	10.52455
Wasi matrix reasoning T score	19	20.00	63.00	38.6842	13.41248
Wasi performance IQ	19	55.00	126.00	83.2632	17.82895
Vineland composite	89	20.00	143.00	55.4719	18.86561
Vineland communication	89	21.00	89.00	49.0899	14.71212
Vineland daily living skills	89	21.00	100.00	60.9101	20.07183
Vineland socialization	89	17.00	83.00	54.5056	14.95913
Vineland motor skills	50	21.00	117.00	73.0400	17.57080
Number of hospitalization	113	0.00	33.00	1.9292	3.86772

**Table 2:** Descriptive parameters of categorical variables.

**Categorical variables**	**Total *N***	**Variable outcome *N***	**Valid %**
Gender (male)	113	92	81.4
Had seizures	113	18	15.9
Currently taking medication	113	57	50.4
Atypical ASD	113	26	23.0
Maternal smoking in pregnancy	99	30	30.3
Maternal medication.n in pregnancy	102	48	47.1
GSTA1 (CC)	112	62	55.4
GSTM1 (active)	112	46	41.1
GSTT1 (active)	112	84	75.0
GSTP1 (IlleIlle)	111	19	17.1

We performed the multiple regression analysis, with each genotype as a predictor, and clinical characteristics (ADI-R scores, Vineland-II scores, number of hospitalizations, presence of seizures during life, and taking psychiatric medication during life) controlling for other genotypes and age. It was shown that the GSTA1^*^CC genotype was a predictor of a lower ADI-R diagnostic communication non-verbal (Bnv) score, as well as, of a lower chance of having seizures during life. Also, this analysis showed that the *GSTM1-active* genotype predicted a higher Vineland total score (as well as communication sub score), meaning better functioning. The *GSTM1-active* genotype was also predictive of more hospitalizations for the child with the ASD diagnosis (Table 3).

**Table 3:** Multiple linear or logictis regression predictor effects of genotype on clinical outcomes (controlling for other genotypes and age).

**Gene predictor**	**Outcome**	**Model parameters**	**Predictor effect**
GSTA1	ADI-R_Bnv	Adjusted *R*^2^ = 0.094 *F* = 2.558^*^	β = −0.232^*^
	ADI-R_C	Adjusted R^2^ = 0.224 F = 6.723^**^	β = 0.178^¥^
	Seizures	Cox & Snell *R*^2^ = 0.183 Nagelkerke *R*^2^ = 0.310 X^2^ = 22.192^**^	Exp(B) = 0.243^*^
GSTM1	VIN	Adjusted *R*^2^ = 0.491 *F* = 17.578^**^	β = 0.160^*^
	Hospitalizations No	Adjusted *R*^2^ = 0.069 *F* = 2.618^*^	β = 0.236^*^

* *< 0.05*;

** *< 0.01*;

¥ *= 0.05; ADI-R_Bnv, ADI-R diagnostic score B nonverbal; ADI-R_C, ADI-R diagnostic score C; Seizures, seizures present/not present; VIN, Vineland adaptive composite score; Hospitalizations No, number of hospitalizations*.

Several moderation analyses were conducted with the clinical characteristics (ADI-R and Vineland scores, number of hospitalizations, having seizures during life, taking psychiatric medication during life) as outcomes, maternal use of medication and smoking during pregnancy as moderators, and each GST genotype as a predictor, controlling for other GST polymorphisms and age in each analysis.

We found the significant effect of interaction between GSTA1 polymorphism and medication use during pregnancy and the ADI-R diagnostic D score. If the mother had used medication during pregnancy, the GSTA1^*^CC genotype was significantly predictive of a lower ADI-R D score, while in case the mother had not used any medication during pregnancy, the GSTA1^*^CC genotype was significantly predictive of a higher ADI-R D score. Also, if the mother smoked during pregnancy, the GSTA1^*^CC genotype significantly predicted a decreased risk of the child taking any medication. GSTA1's predictive effect on child taking medication was not present in case the mother had not smoked in pregnancy.

When it comes to GSTM1 polymorphisms, the *GSTM1-active* genotype was predictive of the higher Vineland score (meaning better functioning), only if the mother had not smoked during pregnancy, whereas no predictive effect was noticed if maternal smoking in pregnancy was present (Table 4). Regarding GSTT1 polymorphism, the *GSTT1-active* genotype significantly predicted a decreased risk of the child having seizures only if the mother had taken medication during pregnancy, whereas this effect was not noticed in case the mother had taken no medication during pregnancy (Table 4).

**Table 4:** Moderation analysis (controlling for other genotypes and age).

**Gene predictor**	**Outcome**	**Maternal moderator variable**	**Moderator value: conditional effect of genotype on outcome B (LLCI;ULCI)**	**Model parameters**	**Interaction effect B (LLCI;ULCI)**
GSTA1	ADI-R_D	Medication in pregnancy	Present: −0.837 (−1.446; −0.227)^†^ Not present: 0.591 (0.007; 1.174)^†^	*R*^2^ = 0.167 F = 2.342^*^	−1.427 (−2.275;−0.5801)^†^
	Patient currently taking medication	Smoking in pregnancy	Present:−3.593 (−6.293; −0.892)^†^ Not present:−0.032 (−1.148; 1.085)	Cox&Snell *R*^2^ = 0.273 Nagelkerke *R*^2^ = 0.364 X^2^ = 30.322^**^	−3.561 (−6.592; −0.528)^†^
GSTM1	VIN	Smoking in pregnancy	Present:−4.785 (−16.550; 6.980) Not present: 13.321 (5.501; 21.141)^†^	*R*^2^ = 0.565 *F* = 12.262^**^	−18.106 (−32.263; −3.949)^†^
GSTT1	Seizures	Medication in pregnancy	Present: −2.814 (−5.356; −0.271)^†^ Not present: 1.031 (−1.661; 3.723)	Cox&Snell *R*^2^ = 0.266 Nagelkerke *R*^2^ = 0.463 X^2^ = 30.361^**^	−3.845 (−7.596; −0.093)

* *< 0.05*;

** *< 0.01*;

† *Significant bootstrapping confidence intervals; ADI-R_D, ADI-R diagnostic score D; Seizures, seizures present/not present; VIN, Vineland adaptive composite score; Hospitalizations No, number of hospitalizations*.

As mentioned, WASI was used only in verbal patients, resulting in a low number of children assessed by this scale. Therefore, we have not included the WASI score in the multiple regression analysis, and we explored the association between specific GST genotypes and intellectual functioning only in the bivariate analysis.

A significant association was found between the *GSTP1* genotype status and intellectual functioning, (WASI), with significant associations with matrix reasoning raw and T score, as well as, with the total IQ score (*p* = 0.034, 0.014, and 0.039, respectively). For each one of the WASI sub scores, it was observed that *IleIle* carriers reached the highest score, *IleVal* carriers somewhat lower, and *ValVal* carriers the lowest score. After the Bonferroni correction, significant differences remained for matrix reasoning score (*p* = 0.035) between the *IleIle* and *IleVal* genotype, with the *IleIle* genotype carriers showing higher scores (better intellectual functioning). Also, a significant difference was shown for matrix reasoning T, between the *IleIle* and *IleVal* genotypes (*p* = 0.028), as well as between the *IleIle* and *ValVal* genotypes (*p* = 0.026), where the *IleIle* genotype carriers also had the highest scores. When it comes to the total IQ score, there was a statistically significant difference (*p* = 0.039) between the *IleIle* and *ValVal* genotype, where the *IleIle* genotype carriers had significantly higher total intellectual functioning scores.

The main ANOVA parameters results are shown in Table 5.

**Table 5:** Correlation of GSTP1 genotypes and intellectual functioning.

	**Genotype**	**Mean**	**SD**	**ANOVA**	
				**F**	***p***
WASI block design raw score	*IleIle*	26.67	20.50	0.757	0.486
	*IleVal*	15.77	20.23		
	*ValVal*	5.00	2.83		
WASI block design T score	*IleIle*	47.00	6.93	2.797	0.093
	*IleVal*	40.46	11.47		
	*ValVal*	24.50	3.54		
Matrix reasoning raw score	*IleIle*	28.33	4.62	4.265	**0.034**
	*IleVal*	12.23	8.98		
	*ValVal*	11.50	12.03		
Matrix reasoning T score	*IleIle*	57.33	3.06	5.719	**0.014**
	*IleVal*	37.31	11.13		
	*ValVal*	28.50	12.02		
IQ	*IleIle*	102.67	3.51	4.076	**0.039**
	*IleVal*	83.38	16.36		
	*ValVal*	64.00	12.73		

*The bold values indicates p < 0.05*.

## Discussion

Our results have shown that the GSTA1^*^CC genotype was a predictor of a lower ADI-R diagnostic non-verbal score, which means a lower impairment in non-verbal communication. This score is calculated in children who are non-verbal and it includes rating of pointing to express interest, nodding, head shaking, conventional/instrumental gestures, as well as, lack of varied spontaneous make-believe or social imitative play—spontaneous imitation of actions, imaginative play, and imitative social play. To our knowledge, there are no studies that specifically explored the effect of GST polymorphisms on symptom severity. Furthermore, as already mentioned, Alabdali et al. have shown that a lower GST activity was correlated with the impairment of social responsiveness (51), which supports the associations observed in our study (more active *GSTA1* genotype is associated with a lower impairment in communication).

This study also showed a significant association between the GSTA1^*^CC genotype and the risk of having convulsions during life, in a way that this variant decreased the risk of seizures. Any significant effect of other examined GST polymorphisms on the risk of seizures was not found in our sample. In contrast to our results, a study performed in a Tunisian population recognized carriers of the *GSTM1-null* genotype to be in a higher risk of epilepsy (52). On the other hand, a study done in an Indian population showed a higher risk of epilepsy in individuals with the *GSTT1-null* genotype and a lower risk in *GSTM1-null* carriers (53). Interestingly, neither of these two studies have explored the role of GSTA1 polymorphism. A recent study which investigated the role of all four common GST polymorphisms in the susceptibility to progressive myoclonus epilepsy (PME) showed the potential effect of the *GSTT1-null* genotype on disease development that was even more potentiated in the carriers of combined GSTA1^*^CC/GSTT1-null genotype who exhibited the greatest risk of developing PME (54). This result is in contrast to our finding, where the GSTA1^*^CC genotype has a potential protective effect, probably due to the fact that PME represents a rather specific entity. The literature search therefore shows only a few studies regarding GST polymorphisms and epilepsy, with conflicting results (52–54). To the best of our knowledge, there are no other studies exploring the effect of GST polymorphisms on the risk of developing seizures in a sample of ASD patients.

According to our findings, the *GSTM1-active* genotype predicted a higher Vineland total score (as well as communication score), meaning a better functioning of children with ASD. A very important study performed in 2017 showed a possibility of predicting ASD diagnosis, as well as, the level of adaptive functioning, based on the statistical multiple regression analysis of antioxidant status in ASD patients vs. neurotypical controls (55). The authors measured elements of folate-dependent one-carbon metabolism (FOCM) and transsulfuration (TS), shown to most likely contribute to the genetic and environmental predisposition to ASD (55, 56). Transsulfuration, specifically, is dependent on glutathione, the main factor in intracellular redox homeostasis, and is highly dependent on genetic factors (like polymorphisms), as well as, exposure to environmental factors such as heavy metals, air pollution, and medication (55). The model proved to be distinctive not only for ASD diagnosis in comparison to neurotypical children, but, when GSSG, tGSH/GSSG, Nitrotyrosine, Tyrosine, and fCysteine were used as inputs, the model was also highly predictive for the level of adaptive functioning, measured by Vineland-II. Our finding which shows that the *GSTM1-active* genotype was associated with a better adaptive functioning, is in line with the previously mentioned study.

The *GSTM1-active* genotype was also predictive of more hospitalizations for the child with an ASD diagnosis. In relation to that, it is important to note that, most of the hospitalizations are in fact for diagnostic purposes, since children from all over the country are assessed at our Institute. Those hospitalizations are mostly needed when there is a difficulty of establishing a diagnosis, and it might mean that children with a better adaptive functioning need a more detailed diagnostic process in order to do that and make a distinction from typically developing children.

In the moderation analysis, we found a significant effect of interaction between the *GSTA1* genotype and medication use during pregnancy, when it comes to predicting the ADI-R diagnostic D score. The GSTA1^*^CC genotype was significantly predictive of the lower ADI-R D score in the case of maternal medication use during pregnancy, whereas the same genotype was predictive of a higher ADI-R D score if maternal medication use was not present during pregnancy. Also, if the mother smoked during pregnancy, the GSTA1^*^CC genotype significantly decreased the risk of child taking medication. That effect was not present in case the mother did not smoke during pregnancy.

In our previous research, the use of any medication during pregnancy significantly increased the risk for ASD in comparison to the healthy controls. Also, there was a significant interaction of the GST genotype with medication use during pregnancy, predictive of ASD risk only in carriers of *GSTM1-null*, as opposed to carriers of *GSTM1-active* (9). It might mean that a lack of GSTM1 activity increases the risk of ASD development in children with intrauterine exposure to medication, while a more active *GSTA1* variant does not lead to the increased risk for ASD *per se*, but in persons with ASD, leads to an earlier symptom presentation and later development of language. Interestingly, if there is already a predisposition for ASD, and the child with the GSTA1^*^CC genotype was exposed to medication during pregnancy, then it leads to the later recognition of autism. It might be hypothesized that another exposure, maybe to a factor that is also a substrate for GSTA1, since GSTs are known for their substrate promiscuity (57), could possibly lead to earlier manifestation of symptoms and later language development; however, only if it is not metabolized in the same extent in the presence of other GSTA1 substrates, such as medication during pregnancy. This might also be a plausible explication for the finding that the GSTA1^*^CC genotype significantly decreases the risk of a child having medication only if the mother smoked during pregnancy. Another possible explanation might be in the fact that GSTA1, together with some other enzymes involved in detoxification, seems to not exhibit the maximal catalytic capacity during intrauterine development, since it has been shown that maturation of certain liver functions, including detoxification, continues months after birth (58).

The significant moderation was also found for GSTM1 polymorphism, namely, the *GSTM1-active* genotype was predictive for the higher Vineland score, meaning a better functioning, only if the mother did not smoke during pregnancy. This finding might be explained by the potential effect of exposure to other factors as well, other than smoking during pregnancy. The *GSTM1-active* genotype contributes to antioxidant capacity and might be regarded as protective against the effects of oxidative stress on ASD presentation, but at a certain point, the defense system might be overwhelmed, leading to oxidative damage and a more pronounced impairment in children with ASD if the child was prenatally exposed to several risk factors (smoking during pregnancy and another potential risk factor).

The *GSTT1-active* genotype significantly predicted a decreased risk of the child having seizures only if the mother had taken medication during pregnancy, whereas this effect was not noticed in case the mother had no medication during pregnancy. As already mentioned, a higher risk of epilepsy has been shown in *GSTT1-null* carriers (53), while another study showed that GSTA1^*^CC/GSTT1-null carriers had the greatest risk of developing PME (54). Both studies showed the protective effect of the *GSTT1-active* genotype with respect to the development of epilepsy. In our study, the protective effect was only observed if the mother was taking medication during pregnancy, which might be possibly associated to the role of GSTT1 in the reactions of bioactivation (57). In our study, the medication that mothers used was for both pregnancy- (progesterone, tocolytics) and health-related issues (mostly antibiotics or antihypertensive therapy). In both cases, it might be said that the child is in a more vulnerable state. It might be speculated that in our sample of children with ASD, the protective effect of the *GSTT1-active* genotype, in terms of a decreased susceptibility to seizures, is exhibited only in high-risk children.

As it was already mentioned, WASI was done only in verbal children in our sample, which comprised a rather small subsample of 19 patients. Accordingly, we have performed a bivariate logistic regression analysis for association between the *GST* genotypes and WASI scores. An interesting finding was that the GSTP1^*^IleIle genotype carriers had a significantly higher total IQ score in comparison to *ValVal* genotype carriers. It might be concluded that the *IleIle* genotype was somewhat protective. A recent case control study that explored the GST activity instead of GST polymorphisms, as well as, mercury and lead levels in the peripheral blood of children with ASD and the healthy controls has shown significantly higher lead and mercury levels, together with a significantly lower GST activity in persons with ASD. Also, this study has shown that these factors were correlated with social responsiveness impairment, as well as clinical symptoms severity (measured by CARS) (51).

A study which explored the interaction of GSTP1, GSTM1, and GSTT1 polymorphisms and prenatal exposure to p,p′-DDT and its effect on cognitive abilities has found it to be significant (33). It was a cohort study done in 326 children. At birth, the concentration of p,p′-DDT was measured, and an assessment of cognitive functioning was done in their fourth year. The pp-DDT concentration was inversely correlated to general cognitive abilities, memory, verbal abilities, and executive functioning, but only in children who had at least one GSTP1 *Val* allele. There were no significant correlations of pp-DDT exposure and cognitive functioning in relation to GSTM1 and GSTT1 polymorphisms (33). This finding is in agreement with the finding from our study, where the carriers of at least one *Val* allele had lower scores of intellectual functioning. Hence, it might be suggested that the GSTP1 polymorphism, although not associated to the ASD risk, is actually potentially connected to the risk of cognitive impairment, probably in interaction with certain factors which were not explored in our study. This finding should be explored further using multivariate analysis in larger samples.

There are several limitations to our study. The sample size is small, therefore, limiting the possibility of including more variables in the analyses. Also, Vineland-II was done only in non-verbal, while WASI was applied only in verbal patients. It would be methodologically better if both Vineland-II and WASI were applied in all participants. By using WASI only in verbal participant, the possibility of exploring the effect of genotypes was limited to bivariate analysis only. Further studies in larger samples should be done in order to explore the possible associations in a broader extent. Another limitation is the lack of detailed information regarding seizures. The question was referred to only having or not having seizures during life, but not the number of seizures, their clinical presentation etc.

On the other hand, our study explored both genetic and environmental factors and their effect on the clinical and intellectual/adaptive characteristics in ASD patients. To our knowledge, this is the first study to specifically explore the GST polymorphisms in relation to ASD symptoms and adaptive functioning impairment severity. In future studies, other environmental factors should be taken into account as well, such as exposure to heavy metals.

## Conclusion

Autism spectrum disorders are a group of heterogeneous and complex developmental disorders. The complexity might be seen in both the genetic and environmental etiological factors. One of the possible mechanisms that could explain a part of both genetic and environmental burden is oxidative stress. Besides the effect that these factors might have on the specific risk of developing ASD (the dichotomy ASD/not ASD), there is also a possible complexity of interactions between the genetic and environmental factors influencing the level of symptoms and impairment in people that have developed an autism spectrum disorder. The result of this study show that these interactions might influence other factors important for overall impairment in ASD, such as cognitive functioning and the risk of developing seizures. Future studies, in larger samples, are needed in order to determine and clarify these interactions, as well as, examination of other possible environmental risk factors.

## Data Availability Statement

The raw data supporting the conclusions of this article will be made available by the authors, without undue reservation.

## Ethics Statement

The studies involving human participants were reviewed and approved by Ethics Committee of the Institute of Mental Health, Belgrade, Serbia. Written informed consent to participate in this study was provided by the participants' legal guardian/next of kin.

## Author Contributions

VM-M, MP-M, MP-E, TP, and TS designed the study. TP, MM-V, and VM-M performed the statistical analysis. VM-M, MP-M, MM-V, and MD recruited and screened the participants. MP-M diagnosed the patients. AS-R performed the genetic analyses. VM-M, MP-M, MP-E, AS-R, ME, and MM-V performed the literature search and wrote the manuscript. TP and TS gave critical comments to the manuscript. All authors contributed to the article and approved the submitted version.

## Conflict of Interest

The authors declare that the research was conducted in the absence of any commercial or financial relationships that could be construed as a potential conflict of interest.
